# The Efficacy of IgM-Enriched Immunoglobulin (eIg) Administration for Treatment of Sepsis and Septic Shock in Adult Surgical Patients: A Single-Center, Retrospective, Observational Study

**DOI:** 10.3390/jcm15041526

**Published:** 2026-02-14

**Authors:** Serena Spanò, Gabriella Licitra, Giada Cucciolini, Etrusca Brogi, Rita Martinelli, Francesco Cundari, Maria Giovanna Curci, Federico Coccolini, Stefano Busani, Giorgio Berlot, Mattia Bixio, Gianni Biancofiore, Francesco Corradi, Francesco Forfori

**Affiliations:** 1 Department of Anesthesia and Intensive Care, Azienda Ospedaliero Universitaria Pisana, 56126 Pisa, Italyfrancesco.forfori@unipi.it (F.F.); 2 Intensive Care Unit, ASST Grande Ospedale Metropolitano Niguarda, 20162 Milan, Italy; 3 General, Emergency and Trauma Surgery Department, Pisa University Hospital, 56100 Pisa, Italy; 4 Anesthesia and Intensive Care Department, University Hospital of Modena, University of Modena, 41125 Reggio Emilia, Italy; 5 Department of Anesthesia and Intensive Care, Ospedale Policlinico San Martino, 16132 Genova, Italy; 6 Department of Transplant Anesthesia and Critical Care, University School of Medicine, 56126 Pisa, Italy

**Keywords:** septic shock, sepsis, surgical patients, IgM-enriched immunoglobulin, eIg, IgGAM (Pentaglobin^®^), sepsis dysfunction, mortality, ICU, average treatment effect (ATE), propensity weighting score analysis

## Abstract

**Background**: Surgical sepsis, particularly secondary peritonitis, is a leading cause of ICU admissions, with mortality rates reaching 40%. In recent decades, several adjuvant therapies have been proposed in addition to standard of care to modulate the inflammatory response and support organ function. In our study, we aimed to evaluate the efficacy of IgM-enriched immunoglobulin (eIg) treatment on outcome of adult surgical patients with sepsis and septic shock. **Methods**: A single-center, retrospective, observational study was conducted from January 2016 to December 2019 in the Intensive Care Unit of Pisa University Hospital. Patients with sepsis or septic shock resulting from primary or postoperative infections undergoing surgical source control were included. The primary outcome was to investigate the impact of eIg administration on in-hospital mortality. The secondary outcomes were the ICU length of stay, days of ventilation, and vasoactive drug administration. A propensity score through inverse probability weighting was used to control for measured confounding variables. **Results**: A total of 108 patients, categorized into two groups based on whether they received eIg, were included during the study period. Compared to the untreated group, patients who received eIg showed a significant reduction in ICU mortality (ATE −0.17, 95% CI −0.33 to −0.03; *p* = 0.023) and in-hospital mortality (ATE −0.18, 95% CI −0.34 to −0.03; *p* = 0.022). However, the ICU length of stay and the duration of mechanical ventilation were significantly longer in the treated group (ATE + 7.1 days, 95% CI 3.1 to 11.1; *p* = 0.001 and ATE + 4.5 days, 95% CI 1.0 to 7.9; *p* = 0.011, respectively). No other statistically significant differences were observed. **Conclusions**: Despite the significant limitations of its observational nature, our study suggests that administering eIg may reduce ICU and in-hospital mortality in surgical patients with sepsis and septic shock.

## 1. Background

Sepsis is defined as “a life-threatening organ dysfunction caused by a dysregulated host response to infection” [1]. This definition encompasses a wide array of clinical phenotypes, ranging from relatively mild presentations to rapidly evolving forms leading to life-threatening multiple organ dysfunction syndrome (MODS) [2,3].

Despite recent improvements in its recognition and management, the mortality rate still remains high, ranging from 40 to 50% in patients with septic shock [4]. In surgical patients, sepsis most commonly arises from postoperative infections, intra-abdominal infections, or surgical site infections; however, the diagnostic criteria and management principles remain the same as those applied to sepsis in general [5].

The time-honored hypothesis of an initial hyperinflammatory phase followed by immune downregulation, ultimately leading to global immunosuppression [6,7,8], has been replaced by a more dynamic model in which pro- and anti-inflammatory responses coexist simultaneously [9,10,11]. This condition ultimately leads to the immune exhaustion, primarily affecting the adaptative immune response [12,13,14,15].

Over the recent decades, several adjuvant therapies have been proposed in addition to standard of care to modulate the inflammatory response and support organ function [16,17,18].

The physiological rationale for the use of the intravenous immunoglobulins in sepsis and septic shock is based on their pleiotropic functions and their depletion during sepsis [19,20,21,22].

As far as the first point is concerned, it should be recalled that immunoglobulins exert antibacterial and anti-endotoxin properties and modulate the immune response by reacting with cells and molecules in pro- and anti-inflammatory pathways [23,24].

Several lines of evidence support the administration of eIg in septic patients. First, low levels of B cells and antibodies are detected within the first 24 h in non-survivors [25,26,27]. Second, the progression from sepsis toward septic shock is marked by the depletion of endogenous immunoglobulin, suggesting a linear correlation between their kinetic features, the severity of hemodynamic impairment, and a poor outcome [28]. In particular, IgM depletion seems to be associated with a reduced ability to counteract gram-negative infections due to its pivotal role in neutralization and opsonization [29,30,31,32].

Over the last years, several retrospective [33,34] and randomized [35,36,37,38,39,40,41] studies were carried out to assess the effectiveness of immunoglobulin administration in septic patients, leading to encouraging results [42,43,44,45]. However, these investigations are affected by multiple sources of bias, including the heterogeneity of patients enrolled, variability in timing and dosage of administration, differences in baseline immune status, variations in eIg composition, treatment duration, and diagnostic criteria [46,47,48,49]. Consequently, the Surviving Sepsis Campaign (SSC) guidelines recommend against the administration of eIg, as the existing evidence largely fails to satisfy evidence-based medicine (EBM) criteria [1].

Given the ongoing uncertainty regarding the effectiveness of the eIg therapy, we aimed to investigate the association between the eIg treatment and the mortality of adult surgical patients with sepsis and septic shock admitted to our center. Our secondary aim was to evaluate whether eIg administration influenced duration of mechanical ventilation, ICU length of stay (LOS), and the need for vasopressor drugs.

## 2. Materials and Methods

### 2.1. Study Design

This single-center, retrospective, observational study included all adult surgical patients with sepsis or septic shock admitted to the postoperative ICU of Pisa University Hospital between January 2016 and December 2019. The study was carried out in accordance with the Declaration of Helsinki and was approved by the local ethics committee (Comitato Etico Area Vasta Nordovest, CEAVNO, protocol code n. 23079 and date of approval 5th of September 2024). Each participant and/or their relatives was contacted and informed that data would be collected for clinical research purposes and that they had the opportunity to oppose.

Adult patients (18 years of age and older) were eligible if they developed sepsis or septic shock due to postoperative infection or primary infection requiring surgical source control. Sepsis and septic shock were defined according to the Sepsis-3 criteria from the Surviving Sepsis Campaign [50,51].

Exclusion criteria included sepsis or septic shock not amenable to surgical management (i.e., medical sepsis), pregnancy, ICU length of stay < 3 days, and pre-existent hematologic disorders.

All patients received standard care according to institutional protocols and current clinical guidelines. The eIg treatment was administered based on clinical judgment, generally within 24 h of sepsis onset. The dosage was determined according to the manufacturer’s recommended regimen of 0.25 g/Kg of body weight per day for 3 consecutive days.

### 2.2. Data Collection

Data were retrospectively obtained from medical records. For all patients, we collected age, sex, comorbidities, Charlson Comorbidity Index, onset time of sepsis and/or septic shock, hospital and ICU admission/discharge dates, and type of surgical admission (e.g., elective, emergency). The Simplified Acute Physiology Score II (SAPSII) [52] was calculated within the first 24 h of ICU admission, and the Sequential Organ Failure Assessment (SOFA) [51] score was assessed daily.

Details on empiric and targeted antibiotic therapy were included, along with the time of administration and the type and number of surgical procedures performed. Empiric therapy was considered adequate if active against a subsequently isolated pathogen or, in the case of negative cultures, if broad-spectrum antibiotics were administered based on hospital epidemiology, infection site, and individual risk factors. Source control was classified as surgical (e.g., laparotomy or reoperation), or non-surgical (e.g., image-guided drainage or interventional radiology procedures). Daily laboratory tests, microbiological cultures with antibiograms and resistance profiles, and the primary infections sites were also noted.

Multidrug-resistant (MDR) microorganisms were defined as those resistant to at least one agent in three or more antibiotic classes. Particular attention was paid to ESKAPE pathogens, ESBL-producing bacteria (extended spectrum beta lactamases), CRE (carbapenem-resistant Enterobacteriaceae), MRSA (methicillin-resistant staphylococcus aureus), MRSE (methicillin-resistant staphylococcus epidermidis), and VRE (vancomycin-resistant Enterobacteriaceae).

Additional variables included the duration of mechanical ventilation (invasive or non-invasive), use and dosage of vasopressors, inotropes, antibiotics and antifungal agents, steroids, and vitamin C, as well as the need for and duration of continuous renal replacement therapy (CRRT).

For patients treated with eIg, we recorded total dose, timing, duration of treatment, and any discontinuation or adverse effects.

### 2.3. Measurements and Outcomes

The primary outcome was to investigate the impact of eIg use on in-hospital mortality; the secondary outcomes were to evaluate its effect on the length of stay in the ICU, days of ventilation, and vasoactive drug administration.

### 2.4. Statistical Analysis

Patients were divided into two groups (eIg group and no-eIg group) based on whether they received eIg (Pentaglobin^®^, manufactured by Grifols, Barcelona, Spain). Data were reported as mean ± standard deviation (SD) or median and interquartile range [IQR, 25th–75th percentile] depending on the distribution. Normality was assessed using the Shapiro–Wilk test and graphical methods. Differences between groups were analyzed using Student’s *t*-test or the Wilcoxon rank-sum test for continuous variables, as appropriate, while categorical variables were compared using the chi-square test or Fisher’s exact test if the expected cell count was less than 5.

We estimated crude mortality in the two groups using the Kaplan–Meier methods and compared survival distributions with the log-rank test.

To explore baseline illness severity, we performed a strip plot of baseline SAPS II values at ICU admission stratified by eIg treatment (yes/no) and hospital mortality (survivors/non-survivors).

Due to the observational nature of the study, we employed a propensity score-inverse probability of treatment weighting analysis with regression adjustment (IPTWRA). This approach, retaining all observations by assigning weights based on the propensity score, can mitigate bias from potential confounders that might be associated with the choice to administer eIg, as well as with clinical outcomes. It accounts for differences in baseline characteristics mimicking randomization effects, like a randomized controlled trial. However, substantial differences exist as the propensity score addresses only measured confounders and not the unmeasured ones. This estimator has the advantage to incorporate the estimated stabilized IPTW and has the feature to be double robustness. Two models are specified (i.e., the model for the outcome and the model for the treatment), and even if one of the models is mis-specified, the estimator is still consistent.

Specifically, the inverse probability of treatment weighting (IPTW) method consists of two steps. First, the propensity score was estimated based on a patient’s likelihood of receiving the treatment in the study, considering their baseline characteristics, and weights were assigned as its inverse. Applying these weights created a pseudo-population in which confounders were more evenly distributed between groups, approximating the conditions of a randomized controlled trial. IPTW using the propensity score creates weights based on the propensity score. In the weighted sample, the distribution of measured baseline covariates will be the same in treated participants as in control participants. Thus, the presence of confounding is removed by weighting, and outcomes can be compared directly between the treated and control participants in the weighted sample.

The variables used for building our model included: site of infection, lactate (>2 mmol/L), procalcitonin (PCT > 0.5 ng/mL), the use of adjuvant therapies, and multidrug-resistant (MDR) organism positivity as categorical variables, as well as the Charlson Comorbidity Index (CCI), SAPS II, and SOFA scores as continuous variables.

To assess covariate balance after applying the estimator, we conducted an overidentification test to validate the results obtained from the standardized difference calculation. In line with the commonly accepted rule of thumb, a standardized difference below 0.10 was considered indicative of adequate balance and reduced sensitivity to model misspecification. However, the overlap was evaluated separately to ensure the reliability of estimated effects.

As the proportion of missing data was below 5%, no imputation was applied and a complete case analysis was carried out. Statistical tests were two-sided, with *p* < 0.05 considered significant.

Statistical analysis was performed using the software Stata, version 17.0. The IPTWRA was computed using the t effects suite. A 5% significance level was used. The design of this study followed the Strengthening in Reporting of Observational Studies in Epidemiology (STROBE) guidelines (Supplemental Material, Table S1) [53].

## 3. Results

### Baseline Characteristics

**Figure 1:**
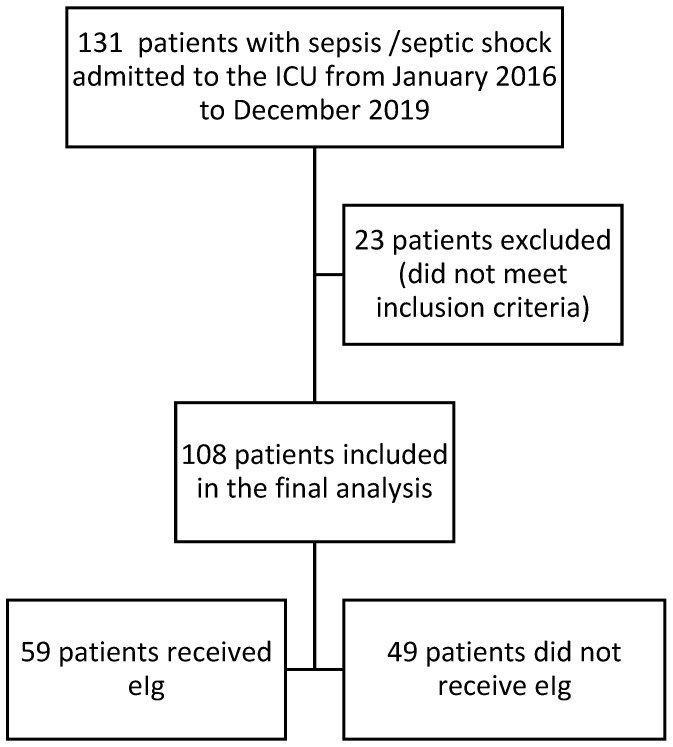
Flow diagram of the population included.

During the study period, 131 patients with sepsis or septic shock were admitted to the ICU (Figure 1). Of these, 108 patients met the inclusion criteria and were included in the analysis, while 23 were excluded due to non-surgical (medical) sepsis. At the admission eIg, 51 pts (52%) had already septic shock, and 46 (48%) pts had sepsis. A total of 7 pts developed sepsis, and 4 pts developed septic shock after the ICU admission. Postoperative sepsis following elective surgery accounted for 81 of cases (75%), while the remaining 26 (25%) were related to the Boerhaave syndrome or secondary peritonitis (e.g., bowel perforation due to inflammatory bowel disease). Patients were divided into two groups based on the administration of eIg (Pentaglobin, manufactured by Grifols, Barcelona, Spain): the eIg group (n = 59) and the standard treatment (no-eIg) group (n = 49).

Both groups had a similar median age of approximately 70 years (*p* = 0.134), and most patients were male and overweight. The Charlson Comorbidity Index was comparable, with a median score of 5 in both groups, indicating a moderate to severe burden of chronic illness (*p* = 0.553). As expected, the abdomen was the most frequent site of infection (80.6% overall), and the disease severity, as measured by SOFA and SAPS II scores, was comparable across groups. A detailed summary of baseline characteristics prior to weighting is provided in Table 1. Lactate levels were slightly higher in the eIg group (2.3 [1.6–3.1] mmol/L) compared to the no-eIg group (1.9 [1.2–2.9] mmol/L), with a statistically significant difference (*p* = 0.052) that appears to have limited clinical relevance.

Procalcitonin levels were also comparable between the two groups (*p* = 0.554), although the eIg group exhibited a higher median and a wider interquartile range (4.2 [0.9–24] vs. 2.6 [0.9–14]). When analyzed as categorical variables, lactate showed a statistically significant difference between groups (*p* = 0.047), whereas procalcitonin did not (*p* = 0.733).

The median ICU stay was 12 days [IQR 6–21] in the eIg group and 9 days [IQR 5–12] in the no-eIg group (*p* = 0.003). At ICU admission, 95.4% of patients required mechanical ventilation, and 82.4% received vasopressor therapy. The proportion of patients needing invasive respiratory support was comparable between groups (*p* = 0.111); however, its duration was longer in the eIg group, with a median of 6 days [IQR 3–14] versus 3 days [IQR 2–7] in the eIg group. Inotropic support was less frequently used overall but was more common in the eIg group. Dobutamine use showed a marginal difference (*p* = 0.086), while levosimendan was administered significantly more often—25.4% in the eIg group versus 10.2% in the no-eIg group (*p* = 0.043). No significant difference was observed in the duration of vasoactive therapy (*p* = 0.120).

Steroids were widely used, but their administration was significantly more frequent in the eIg group (72.9%) compared to the no-eIg group (46.9%) (*p* = 0.006). No other statistically significant differences were observed in the use of remaining adjuvant therapies.

Empiric antibiotic therapy was appropriate in most cases, according to culture samples collected. Blood cultures were negative in 30% of patients. Gram-negative bacteria were the predominant pathogens, with *Pseudomonas aeruginosa*, *Acinetobacter baumannii*, *Escherichia coli*, and *Klebsiella pneumoniae* being most frequently isolated. Among gram-positive organisms, *Enterococcus faecium*, *Enterococcus faecalis*, and *Staphylococcus* spp. were the most common.

Forty-nine patients died: 23 in the ICU and 26 after transfer to other hospital wards. No adverse reactions to eIg were observed. The treatment was initiated within 24 h of sepsis or septic shock onset and was administered over a 3-day course without unexpected interruptions.

**Figure 2:**
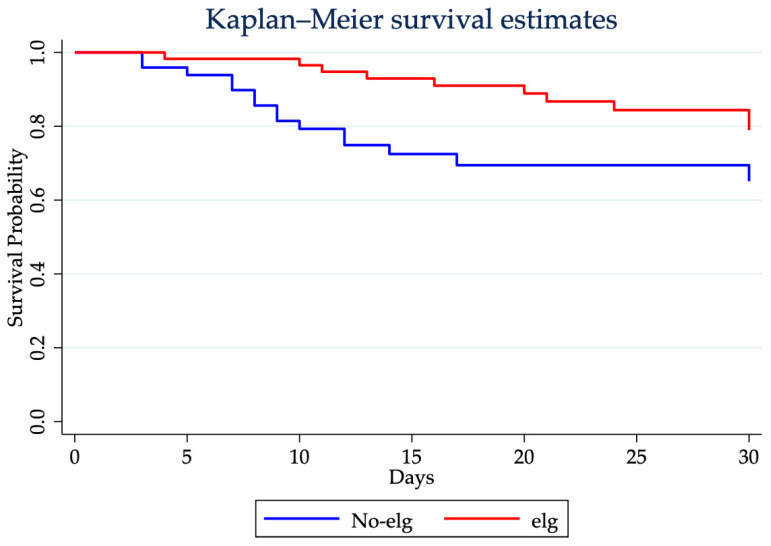
Kaplan–Meier survival curves for patients who did (red line) and did not (blue line) receive eIg during the first 30 days of the ICU stay. Patients treated with eIg showed a higher survival probability throughout the observation period compared with the no-eIg group, with early separation of the curves that persisted until day 30.

Figure 2 displays the Kaplan–Meier curves comparing 30-day mortality between the two groups. The log-rank test indicated a statistically significant difference (*p* = 0.037).

**Figure 3:**
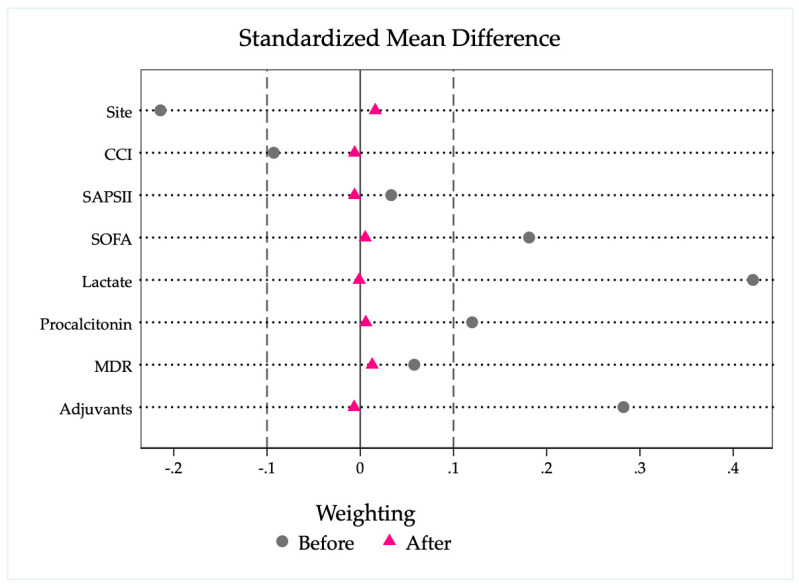
Standardized mean differences (SMDs) for all patient characteristics included in the propensity score model before and after inverse probability weighting with regression adjustment (IPWRA). The SMD quantifies the difference in means between groups in units of standard deviation. After weighting, all covariate imbalances were reduced to <10% (dashed line), suggesting adequate covariate balance.

Figure 3 presents the assessment of covariate balance before and after weighting. Variables included in the model—selected based on the directed acyclic graph (DAG) (Supplemental Figure S1)—demonstrated satisfactory balance, with standardized mean differences below 0.1 and variance ratios close to 1. The propensity score overlap between treatment groups was acceptable, as shown in (Supplemental Figure S2).

**Figure 4:**
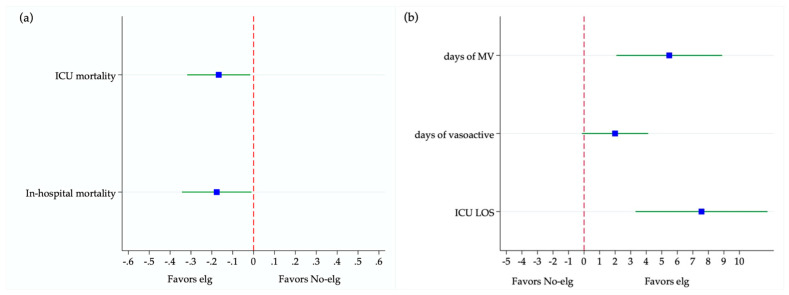
Forest plot showing the average treatment effects (ATE) estimated using the inverse probability weighting with regression adjustment (IPWRA). (**a**) Treatment with eIg was associated with a reduction in ICU and in-hospital mortality compared with standard treatment (no-eIg). (**b**) Treatment with eIg was associated with longer duration of mechanical ventilation, vasoactive support, and ICU length of stay. Point estimates (blue squares) represent ATEs, and horizontal lines indicate 95% confidence intervals. Values indicating effects favoring eIg are reversed between the two panels because the ATE has opposite signs across outcomes. In (**a**), values left of the red dashed line favor eIg; in (**b**), values left of the line favor no- eIg.

Table 2 reports the average treatment effect (ATE) estimated using the IPWRA. Patients who received eIg had lower ICU and hospital mortality compared to those who did not. The ATE showed a reduction of 17 percentage points in ICU mortality and 18 percentage points in hospital mortality (*p* = 0.023 and *p* = 0.022, respectively). Conversely, the no-eIg group had a shorter ICU length of stay and required fewer days of mechanical ventilation. No statistically significant difference was observed in the duration of vasoactive support. A graphical representation of the IPWRA results is shown in Figure 4.

**Figure 5:**
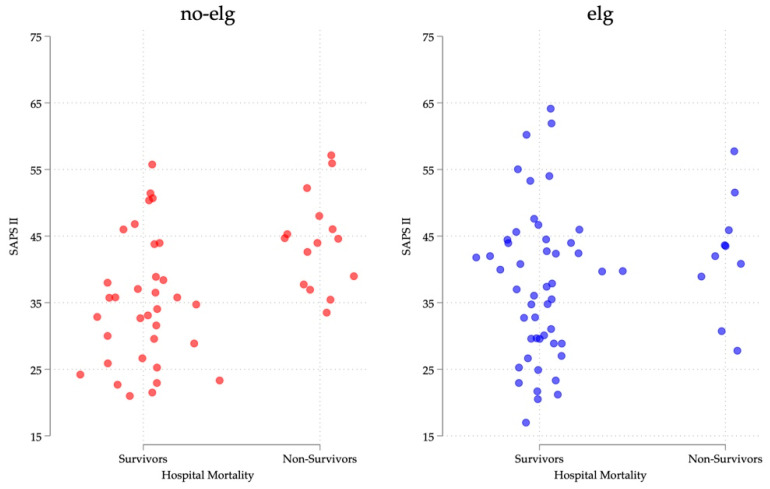
Distribution of baseline SAPS II values according to the eIg treatment status and hospital mortality. The graphical distribution appears slightly shifted towards higher values in the eIg group; however, no statistically significant difference was detected (Wilcoxon rank-sum test, *p* = 0.83). Stripplot of baseline SAPS II values at ICU admission stratified by treatment (yes/no) and hospital mortality (survivors/non-survivors).

Figure 5 displays the distribution of baseline SAPS II values according to eIg treatment status and hospital mortality. The graphical distribution appears slightly shifted toward higher values in the eIg group; however, no statistically significant difference was detected (Wilcoxon rank-sum test, *p* = 0.83).

## 4. Discussion

Despite decades of studies aimed at finding a possible new treatment strategy for modulating the sepsis-associated immune dysfunctions by targeting specific mediators, timely administration of antibiotics, and effective source control still remain the cornerstone of the septic shock management [1,54,55]. Beyond infection and the host immune response, patient outcomes are influenced by multiple factors, including nutritional status, muscle catabolism, age, comorbidities and frailty, ICU and hospital length of stay, multidrug-resistant pathogens, and viral reactivation [56]. The clinical course is often characterized by prolonged ICU stays, frequently culminating in death among patients who survive the initial insult. We conducted a retrospective study to evaluate the efficacy of enriched immunoglobulins (eIg) on outcomes in surgical patients with sepsis. The administration of eIg has been proposed as a potentially valuable adjunctive therapy, both during the early hyperinflammatory phase and in later stages, characterized by immunoparalysis [24]. In our cohort, patients receiving eIg presented a higher survival rate compared with standard care alone. Notably, divergence in survival curves became evident within the first 5 days following sepsis diagnosis. Given that the appropriateness of antibiotic therapy was comparable between the two groups, it is reasonable to hypothesize that this early survival benefit may be attributable to eIg administration during this critical time window. Furthermore, among survivors, the ICU LOS and mechanical ventilation duration were longer in the eIg group. The concordant direction of these outcomes is suggestive of differences in baseline risk at ICU admission, in line with the SAPS II strip plot showing a tendency toward higher values among eIg-treated survivors, although the difference was not statistically significant. These findings are also in line with the heterogeneous literature. As summarized by Kakoullis et al., the ICU LOS has been evaluated across several studies with often inconsistent results, and most reports did not detect statistically significant differences between treated and control groups [57]. Nevertheless, some studies described longer ICU stays in treated patients, including the randomized trial by Rodríguez et al. (all comparisons *p* > 0.05) and the study by Yavuz et al., which reported a significantly longer ICU LOS in the IgM-enriched IVIG group (*p* = 0.002) [36,58]. Consistently, meta-analytic evidence, including that presented by Cui et al., does not support a reproducible reduction in the ICU LOS with IgM-enriched immunoglobulins [59].

Additionally, the ICU LOS is a heterogeneous endpoint that may be influenced by factors not fully captured in observational datasets, leading to residual confounding (e.g., the feasibility or difficulty of achieving timely and effective source control). The ICU LOS and mechanical ventilation duration are also sensitive to survival dynamics: preventing early deaths can prolong the observed ICU stays among survivors, thereby attenuating the artificially shortened LOS seen in cohorts with higher early mortality. For these reasons, there is an increasing interest in composite time-to-recovery outcomes such as ventilator-free days and ICU-free days, which may provide a more informative summary of recovery and ICU resource use than traditional measures such as the ICU LOS alone; they are also less sensitive to early mortality.

The clinical efficacy of the eIg infusion in surgical sepsis and septic shock has been demonstrated in previous studies [38,60]. Rodriguez et al. showed that adequate antibiotic therapy combined with immunoglobulin administration improved prognosis, reducing 30-day mortality by 25% in surgical patients with abdominal infections [36]. Notably, the positive effects of eIg on the outcome of septic shock patients are not limited to surgical populations. In 2014, Cavazzuti et al. performed a retrospective observational study evaluating the association between IgM treatment and 30-day mortality in 168 septic patients from medical and surgical departments, demonstrating a 20% reduction in 28-day mortality among patients who received eIg (25.4% vs. 45.8%; OR 0.35, 95% CI 0.14–0.85; *p* = 0.021) [34]. IgM-enriched immunoglobulin therapy was also associated with an increase in ventilator-free days and ICU-free days. Perrella et al. conducted a retrospective study evaluating the efficacy of pentameric IgM in adult patients admitted to the ICU for sepsis or septic shock following major abdominal surgery [61]. Patients treated with pentameric IgM in addition to antibiotics were compared with those receiving antibiotic therapy alone. No significant differences were observed between groups in terms of microbiological isolates, inflammatory markers, lactate levels, or mortality. Interestingly, an early clinical response within 48 h was more frequently observed in the antibiotic-only group. Overall, the study did not demonstrate a clear clinical benefit of pentameric IgM administration in this specific surgical population.

Berlot et al. demonstrated in 355 septic shock patients treated with eIg in addition to standard care that (a) mortality reduction was associated with early administration, (b) this effect was also observed in patients with sepsis-related multidrug-resistant (MDR) infections, and (c) the association was even more pronounced in the subgroup of surgical patients [62].

Regarding the composition of available intravenous immunoglobulin preparations and their related clinical effects, a recent meta-analysis including 19 studies by Cui et al. concluded that the administration of eIg—an IgA- and IgM-enriched immunoglobulin preparation (Pentaglobin^®^, manufactured by Grifols, Spain)—in septic shock patients was associated with lower mortality and shorter duration of mechanical ventilation compared with other preparations containing lower concentrations of these immunoglobulins [59]. The effectiveness of eIg has been further supported by a more recent meta-analysis including 31 randomized controlled trials with a low risk of bias [63].

The relative proportions of immunoglobulins in eIg closely resemble physiological plasma concentrations (IgM 12%, IgA 12%, and IgG 76%) and may confer specific biological advantages. First, IgM is particularly effective against gram-negative infections due to its strong neutralizing and opsonizing capacity, acting as a functional bridge between the innate and adaptive immune responses [64,65]. Second, IgA plays a key role in maintaining the barrier function of the epithelial lining of the pulmonary and intestinal mucosa [66,67,68].

Nevertheless, despite these findings, the current Surviving Sepsis Campaign (SSC) guidelines recommend against the routine use of eIg, mainly due to the lack of randomized clinical trials meeting evidence-based medicine (EBM) criteria [1]. As it appears unlikely that such trials will be conducted in the near future, it may be worthwhile to define pragmatic “rules of engagement” (ROE) aimed at identifying patient subgroups most likely to benefit from eIg therapy.

Based on the above-mentioned studies, several clinical scenarios may be proposed to guide patient selection. First, patients admitted to the ICU within 48 h of septic shock onset appear to be more suitable candidates; within this group, surgical patients seem to derive greater benefit compared with those with septic shock of medical origin. Second, patients whose severity of illness is assessed by integrating baseline comorbidities and the magnitude of the host response to infection, as reflected by composite scores such as the PIRO score, may represent an appropriate target population [69,70]. Third, chronically critically ill patients who survive the initial septic insult but subsequently develop a state of persistent inflammation characterized by progressive muscle wasting, prolonged mechanical ventilation, and immune exhaustion may also be considered as potential candidates for eIg therapy [71]. More recently, a novel scoring system specifically designed to guide eIg administration—the SORRISO score—has been developed and published. Although this score requires validation in larger patient cohorts, it represents a promising and practical bedside tool to improve patient selection for eIg therapy [72].

### Limitations

Despite the overall robustness of our statistical approach, several limitations must be acknowledged. Although inverse probability weighting (IPW) techniques can partially “mimic” randomization, only true randomization can ensure balance across both measured and unmeasured (or unknown) confounders. Propensity score–based methods adjust exclusively for observed variables and cannot account for unmeasured confounding or missing data. Post-estimation diagnostics demonstrated an acceptable, though not optimal, overlap between the weighted groups, suggesting that residual confounding from unmeasured variables may still have influenced the observed outcomes and was therefore not accounted for in the analysis.

Furthermore, the retrospective design of the study and the relatively small sample size limit the statistical power and generalizability of our findings.

Another important limitation is that no immunological biomarkers were assessed. Information regarding patients’ immune status would have been highly valuable both for refining patient selection and for better evaluating treatment effectiveness. Moreover, correlating clinical outcomes with the kinetics of plasma immunoglobulin levels during therapy could have provided additional insight into the relationship between eIg administration and clinical response.

Several studies have demonstrated that immune dysregulation may persist in sepsis survivors due to prolonged immune suppression, leading to hospital readmissions and late mortality [69,70]. In our study, post-discharge follow-up was not available, precluding the assessment of reinfections, readmissions, or late deaths.

## 5. Conclusions

Despite these limitations, our findings—consistent with previously published evidence—suggest that the administration of eIg (Pentaglobin^®^) may be associated with reduced ICU and hospital mortality in surgical patients with sepsis and septic shock. Conversely, in our cohort, the mechanical ventilation duration and ICU length of stay were longer in the eIg group, likely reflecting greater baseline disease severity. The duration of the vasopressor therapy did not appear to be influenced by eIg administration.

With this study, we aim to contribute encouraging prospective study supporting the use of eIg in surgical septic patients. Further studies are warranted to confirm these findings and to better elucidate the role of eIg in sepsis management. We acknowledge the limitations imposed by the small sample size; however, as emphasized by Hernán et al. [73], the objective of observational causal inference is not merely to detect effects, but to provide transparent and reproducible estimates. Even imprecise estimates add to the existing evidence base and may inform future meta-analyses.

## Figures and Tables

**Table 1:** Baseline clinical and demographic characteristics.

Variables	Overall (n = 108)	eIg (n = 59)	No-eIg (n = 49)	*p*-Value	Missing Data n (%)
Demographics and comorbidities					
Age, years, median (IQR)	70 [60.5–77]	69 [60–75]	70 [63–79]	0.134 ^W^	0 (0.0)
BMI (>25)	59 (57.8)	34 (59.7)	25 (55.6)	0.678 ^χ^	6 (5.6)
Sex (female)	47 (43.5)	26(44.1)	21 (42.9)	0.899 ^χ^	0 (0.0)
Charlson Comorbidity Index, median (IQR)	5 [3–7]	5 [3–7]	5 [3–8]	0.553 ^W^	0 (0.0)
Site of infection Abdominal Thoracic Blood and Co.	87 (80.6) 20 (18.5) 1 (0.9)	50 (84.6) 9 (15.3)0	37 (75.5) 11 (22.5) 1 (2.0)	0.267 ^F^	0 (0.0)
Disease severity (admission)					
SOFA, mean (SD)	6.9 (2.7)	7.2 (2.9)	6.6 (2.5)	0.288 ^t^	1 (0.9)
SAPS II, mean (SD)	38 (10.2)	38.2 (10.7)	37.7 (9.6)	0.782 ^t^	0 (0.0)
Lab results					
Lactate, median (IQR)	2 [1.5–3]	2.3 [1.6–3.1]	1.9 [1.2–2.9]	0.052 ^W^	0 (0.0)
Lactate (>2 mmol/L)	51 (47.2)	33 (55.9)	18 (36.7)	0.047 ^χ^	0 (0.0)
PCT, median (IQR)	3.0 [0.9–18.7]	4.2 [0.9–24]	2.6 [0.9–14.0]	0.554 ^W^	0 (0.0)
PCT (>0.5 ng/mL)	96(88.9)	53(89.8)	43(87.8)	0.733 ^χ^	0 (0.0)
Therapeutic interventions					
Mechanical ventilation (yes)	103 (95.4)	58 [98.3]	45 [91.8]	0.111 ^χ^	0 (0.0)
Vasopressor (yes)	89 [82.4]	50 [84.8]	39 [79.6]	0.484 ^χ^	0 (0.0)
Dobutamine (yes)	26 [24.1]	18 [30.5]	8 [16.3]	0.086 ^χ^	0 (0.0)
Levosimendan (yes)	20 [18.5]	15 [25.4]	5 [10.2]	0.043 ^F^	0 (0.0)
Vit C (yes)	45 (41.7)	27 (45.8)	18 (36.7)	0.343 ^χ^	0 (0.0)
Toraymixin (yes)	8 (7.4)	7 (11.9)	1 (2.0)	0.069 ^χ^	0 (0.0)
Steroids (yes)	66 (61.1)	43 (72.9)	23 (46.9)	0.006 ^χ^	0 (0.0)
CPFA (yes)	3 (2.8)	2(3.4)	1 (2.0)	0.671 ^F^	0 (0.0)
CRRT (yes)	27 [25.0]	15 [25.4]	12 [24.5]	0.911 ^χ^	0 (0.0)
Outcome					
Time (vasoactive), days, median (IQR)	3 [1.5 to 5]	4 [2–6]	3 [1–4]	0.120 ^W^	0 (0.0)
Mechanical ventilation (days), median (IQR)	4.5 [1–11]	6 [3–14]	3 [2–7]	0.007 ^W^	4 (3.7)
ICU LOS days, median (IQR)	9.5 [5–17]	12 [6–21]	8 [5–12]	0.003 ^W^	0 (0.0)
ICU death (yes)	23 [21.3]	9 [15.6]	14 [28.6]	0.092 ^χ^	0 (0.0)
Hospital death (yes)	26 [24.8]	10 [17.5]	16 [33.3]	0.094 ^χ^	0 (0.0)

Data are presented as median (interquartile range) for age, Charlson Comorbidity Index (CCI), procalcitonin (PCT), lactatel, use of mechanical ventilation, and administration of vasoactive agents. Other continuous variables are expressed as mean (standard deviation), and categorical variables are reported as count (percentage). The last column shows the incidence of missing data, expressed as count (percentage). Due to the violation of the linearity assumption with log odds of mortality for lactate and procalcitonin, both variables were dichotomized at 2 mmol/L and 0.5 ng/mL, respectively. ^W^ Wilcoxon Rank Sum test, ^χ^ chi-square test, ^F^ Fisher’s exact Test, and ^t^ *t*-test. Abbreviations: BMI: body mass index; ICU: intensive care unit; CRRT: continuous renal replacement therapy; CPFA: coupled plasma filtration adsorption; PCT: procalcitonin; SOFA: Sequential Organ Failure Assessment; SAPS II: Simplified Acute Physiology Score II.

**Table 2:** Estimated average treatment effect (ATE) from IPWRA.

	no-eIg Coef. [95% CI]	eIg Coef. [95% CI]	ATE [95% CI]	*p* Value
Length of Stay in ICU (Days)	9.9 [7.8 to 12.0]	17.1 [13.3 to 21.0]	7.2 [2.8 to 11.7]	0.002
Mechanical Ventilation (days)	5.9 [4.3 to 7.4]	10.5 [7.6 to13.5]	4.7 [1.3 to 8.1]	0.007
Vasoactive Administration (Days)	4.3 [3.1 to 5.5]	6.0 [4.0 to 8.0]	1.7 [−0.6 to 4.0]	0.145
ICU Mortality	0.32 [0.19 to 0.44]	0.15 [0.05 to 0.24]	−0.17 [−0.32 to −0.02]	0.030
In-Hospital Mortality	0.34 [0.20 to 0.48]	0.16 [0.06 to 0.26]	−0.18 [−0.34 to −0.01]	0.038

Because ICU length of stay, days of mechanical ventilation, and vasoactive administration are necessarily positive variables, we used the Poisson option in the outcome model specification. When considering ICU length of stay, the estimated potential outcome mean (POM) of the control level (No eIg) is 9.9 days, while the estimated average treatment effect (ATE) for patients receiving eIg is 7.2 days. Abbreviations: ICU: intensive care unit; No eIg: eIg: intravenous immunoglobulins enriched with IgA and IgM group.

## Data Availability

The original contributions presented in this study are included in the article. Further inquiries can be directed to the corresponding author.

## Supplementary Materials

The following supporting information can be downloaded at: https://www.mdpi.com/article/10.3390/jcm15041526/s1, Figure S1: Directed acyclic graph (DAG) depicting variables of interest (dots) and causal relantionship between the exposure (treatment with eIg) and the primary outcome (ICU mortality), and the baseline covariates that affects this relationship. SOFA = sequential organ failure assessment score. Dot in blue: primary outcome. Green dot: exposure. The relationship of interest in this study is the one connecting both points. In red: predictor variables that affect both the exposure and the outcome in which direct data was available. In light grey: the presence of complications could influence the outcome but not the treatment received, hence it was not adjusted for; Figure S2: The graph displays the estimated density of the predicted probabilities that a patient who received eIg is classified as a no-eIg patient and the estimated density of the predicted probabilities that a patient who did not receive eIg is classified as an eIg patient, after applying stabilized IPTW. Neither distribution shows excessive probability mass near 0 or 1, and the two estimated densities have most of their respective mass in regions in which they overlap. Thus, there is no evidence that the overlap assumption is violated; Table S1: STROBE Statement—checklist of items that should be included in reports of observational studies.

## Abbreviation

ICU: intensive care unit; eIg: intravenous immunoglobulin; ATE: average treatment effects; MODS: multiple organ dysfunctions; SSC: Surviving Sepsis Campaign; EBM: evidence-based medicine; LOS: length of stay; CEAVNO: Comitato Etico Area Vasta Nordovest; SAPSII: Simplified Acute Physiology Score II; SOFA: Sequential Organ Failure Assessment; MDR: multidrug-resistant; ESBL-producing bacteria: extended spectrum beta lactamases; CRE: carbapenem-resistant Enterobacteriaceae; MRSA: methicillin-resistant staphylococcus aureus; MRSE: methicillin-resistant staphylococcus epidermidis; VRE: vancomycin-resistant Enterobacteriaceae; CRRT: continuous renal replacement therapy; SD: standard deviation; IPTWRA: propensity score-inverse probability of treatment weighting analysis with regression adjustment; PCT: procalcitonin; CCI: Charlson Comorbidity Index; STROBE: Strengthening in Reporting of Observational Studies in Epidemiology; IQR: interquartile range; DAG: directed acyclic graph; RCT: randomized control trial; ROE: rules of engagement.
