# Supplementary material for: The Efficacy of IgM-Enriched Immunoglobulin (eIg) Administration for Treatment of Sepsis and Septic Shock in Adult Surgical Patients: A Single-Center, Retrospective, Observational Study
Source: J Clin Med. 2026 Feb 14;15(4):1526. doi: 10.3390/jcm15041526 (PMC12941539; doi:10.3390/jcm15041526)
Supplement: Supplementary file 1 [file jcm-15-01526-s001.zip › jcm-4103964-supplementary.pdf]

Journal of Clinical Medicine

Supplemental Material Table Legend

Supplemental Material Table S1: STROBE Statement—checklist of items that should be included in reports of observational studies.

	Item No.	Recommendation	Page No.	Relevant text from manuscript
**Title and abstract**	1	(*a*) Indicate the study's design with a commonly used term in the title or the abstract	1	Efficacy of IgM-enriched Immunoglobulin (eIg) administration for treatment of sepsis and septic shock in adult surgical patients: a single center, retrospective and observational study
		(*b*) Provide in the abstract an informative and balanced summary of what was done and what was found	2-3	**Methods:** A single-center, retrospective observational study was conducted from January 2016 to December 2019 in the Polyvalent Intensive Care Unit of Pisa University Hospital. Patients with sepsis or septic shock resulting from postoperative or primary infections undergoing surgical source control were included. The primary outcome was to investigate the impact of IgM-enriched immunoglobulin (eIg) administration on in-hospital mortality. The secondary outcome was to evaluate its effect on ICU length of stay, days of ventilation and vasoactive drug administration. Due to the observational nature of the study, propensity score through inverse probability weighting was used to control for measured confounding variables. **Results** A total of one hundred eight patients, categorized into two groups based on whether they received IgM-enriched intravenous immunoglobulin (eIg), were included during the study period. Compared to the untreated group, patients who received IgM-enriched IVIg (eIg) showed a significant reduction in ICU

				mortality (ATE −0.17, 95% CI −0.33 to −0.03; p = 0.023) and hospital mortality (ATE −0.18, 95% CI −0.34 to −0.03; p = 0.022). However, ICU length of stay and duration of mechanical ventilation were significantly longer in the treated group (ATE +7.1 days, 95% CI 3.1 to 11.1; p = 0.001, and ATE +4.5 days, 95% CI 1.0 to 7.9; p = 0.011, respectively). No other statistically significant differences were observed.
**Introduction**				
Background/rationale	2	Explain the scientific background and rationale for the investigation being reported	4-5	Sepsis is a life-threatening condition characterized by organ dysfunction resulting from a dysregulated host response to infection. Current understanding recognizes the simultaneous presence of pro- and anti-inflammatory mechanisms, which can lead to immune exhaustion, particularly affecting the adaptive immune system. Despite advances in management, mortality in septic shock remains high. Intravenous immunoglobulins (eIg) have been proposed as adjunctive therapy due to their pleiotropic immunomodulatory effects, yet their endogenous levels are often depleted during sepsis. Preparations enriched in IgM and IgA (eIg) may offer additional benefits, particularly in supporting antimicrobial defense and mucosal immunity. However, available evidence is limited by heterogeneity in patient populations, treatment protocols, and study design, and guidelines currently discourage routine use of IvIg. This study was conducted to investigate the association between eIg administration and clinical outcomes in adult surgical patients with sepsis and septic shock.
Objectives	3	State specific objectives, including any prespecified hypotheses	7	The primary outcome was to investigate the impact of IgM-enriched (eIg) immunoglobulin use on improving in-hospital and ICU mortality; the secondary outcome was to evaluate its effect

				on the length of stay in the intensive care unit, days of ventilation and vasoactive drug administration.
**Methods**				
Study design	4	Present key elements of study design early in the paper	6-7	This is a single-center, retrospective observational study conducted to evaluate the association between enriched intravenous immunoglobulin (eIg) therapy and clinical outcomes in adult surgical patients with sepsis and septic shock. Given the non-randomized design, potential confounding was addressed using propensity score weighting using Inverse Probability of Treatment Weighting with regression adjustment (IPTWRA), described in detail in the statistical analysis section. This estimator has the advantage to incorporate the estimated stabilized IPTW and has the feature to be double robustness. It allows to write a model for the treatment and a model for the outcome. Even if one of the models (treatment or outcome) is misspecified, it produces robust estimates.
Setting	5	Describe the setting, locations, and relevant dates, including periods of recruitment, exposure, follow-up, and data collection	6-7	Data were retrospectively collected from electronic medical records. All adult patients meeting the inclusion criteria and admitted to the ICU for sepsis or septic shock between January 2016 and December 2019 were included. Exposure to enriched intravenous immunoglobulin (eIg) occurred during the ICU stay, based on clinical judgment and institutional protocols. Patients were followed from ICU admission (baseline) until death or hospital discharge. Data collection and follow-up were completed by the end of hospitalization.
Participants	6	(*a*) *Cohort study*—Give the eligibility criteria, and the sources and methods of selection of participants. Describe methods of follow-up	5-7	Adult patients (>18 years old) were eligible if they developed sepsis or septic shock due to postoperative infection or primary infection requiring surgical source control. Sepsis and septic shock were defined according to the Sepsis-3 criteria from the Surviving Sepsis Campaign Exclusion criteria included sepsis or

		Case-control study—Give the eligibility criteria, and the sources and methods of case ascertainment and control selection. Give the rationale for the choice of cases and controls<br>*Cross-sectional study*—Give the eligibility criteria, and the sources and methods of selection of participants		septic shock not amenable to surgical management (i.e. medical sepsis), pregnancy, ICU length of stay < 3 days, and pre-existent hematologic disorders.
		(*b*) *Cohort study*—For matched studies, give matching criteria and number of exposed and unexposed<br>*Case-control study*—For matched studies, give matching criteria and the number of controls per case	NA	
Variables	7	Clearly define all outcomes, exposures, predictors, potential confounders, and effect modifiers. Give diagnostic criteria, if applicable	6-9, supplemental figure e1	The primary outcome was to evaluate the impact of IgM-enriched intravenous immunoglobulin (eIg) administration on in-hospital and ICU mortality. The secondary outcome was to assess its effect on the length of stay in the intensive care unit, days of ventilation, vasoactive drug administration. All patients in the eIg group received a Immunoglobulin infusion which started generally within 24h of sepsis onset, and the dose was calculated according to the therapeutic scheme recommended by the productor.<br>The effect of eIg on the primary outcome was then estimated using Inverse Probability of Treatment Weighting with regression adjustment (IPTWRA). Covariates entered into the model were as follows: site of infection, Charlson Comorbidity Index, SAPSII, Sofa, Lactate, procalcitonin, adjuvants, MDRD+.
Data sources/ measurement	8*	For each variable of interest, give sources of data and details of methods of assessment (measurement). Describe comparability of assessment methods if there is more than one group	6-7	Data were extracted retrospectively from electronic medical records. SAPS II was calculated within 24 h of ICU admission. SOFA score, PCT, lactate, and other laboratory data were recorded daily. Antibiotic and adjunctive therapies, surgical interventions, culture results, and

				duration of organ support therapies were also collected. Exposure to IgM-enriched IVIg (eIg) was recorded with dose and timing.
Bias	9	Describe any efforts to address potential sources of bias	7-9	To mitigate bias associated with non-random treatment allocation, we applied inverse probability of treatment weighting with regression adjtment (IPTWRA) based on propensity scores, allowing for adjustment of measured baseline differences between treated and untreated patients. This method retains all observations while creating a pseudo-population in which treatment assignment is independent of observed covariates. Although this approach adjusts for measured confounders, residual confounding due to unmeasured variables remains possible. Selection bias was minimized by including all consecutive eligible patients over a four-year period, and follow-up bias was unlikely, as outcomes were assessed up to hospital discharge or in-hospital death. The IPTW-RA is a double-robust estimator that combines inverse probability of treatment weighting (IPTW) with regression adjustment (RA). This approach ensures consistent estimates even if one of the two models—either the treatment model (IPTW) or the outcome model (RA)—is misspecified. Specifically, if the regression model is incorrect but the propensity score model is correctly specified, the IPTW-RA corrects the estimates. Conversely, if the outcome model is correctly specified, the estimator remains consistent regardless of misspecification of the treatment model.
Study size	10	Explain how the study size was arrived at	NA	No formal sample size calculation was performed. All eligible patients admitted during the study period were included to maximize representativeness and minimize selection bias.

Quantitative variables	11	Explain how quantitative variables were handled in the analyses. If applicable, describe which groupings were chosen and why	Table 1, 7-9	Continuous variables included in the propensity score model were the SOFA score, SAPS II, and Charlson Comorbidity Index. Lactate and procalcitonin were dichotomized using the Sepsis-3 cutoff for lactate (>2 mmol/L) and the standard threshold for procalcitonin (>0.5 ng/mL). This categorization improved covariate balance between treatment groups. Variable selection for the model was guided by a directed acyclic graph (DAG) developed from clinical knowledge and evidence from the literature.
Statistical methods	12	(*a*) Describe all statistical methods, including those used to control for confounding	7-9	Differences between groups were assessed using t-tests, Wilcoxon rank-sum tests, chi-square, or Fisher's exact test. Crude mortality was analyzed with Kaplan-Meier curves and log-rank tests. Propensity score-based inverse probability of treatment weighting rith regression adjustment (IPTWRA) was applied to adjust for confounding. Covariate balance was assessed using standardized mean difference and variance ratio. Overlap was visually checked. Average treatment effect (ATE) was estimated using IPTWRA. A complete case analysis was conducted due to percentage close to 5% missing data. All analyses were performed in Stata 17.0.We performed a complete case analysis, excluding patients that had any missing data on the selected variables to construct the propensity score.
		(*b*) Describe any methods used to examine subgroups and interactions	NA	
		(*c*) Explain how missing data were addressed	9	As the overall proportion of missing data was below 5%, no imputation was performed, and a complete-case analysis was conducted. A detailed overview of missing data is presented in

				Table 1; however, all variables included in the statistical analysis were complete, with no missing values.
		(*d*) *Cohort study*—If applicable, explain how loss to follow-up was addressed<br>*Case-control study*—If applicable, explain how matching of cases and controls was addressed<br>*Cross-sectional study*—If applicable, describe analytical methods taking account of sampling strategy	NA	
		(e) Describe any sensitivity analyses	NA	
**Results**				
Participants	13*	(a) Report numbers of individuals at each stage of study—eg numbers potentially eligible, examined for eligibility, confirmed eligible, included in the study, completing follow-up, and analysed	Figure 1	
		(b) Give reasons for non-participation at each stage	Figure 1	
		(c) Consider use of a flow diagram	Figure 1	
Descriptive data	14*	(a) Give characteristics of study participants (eg demographic, clinical, social) and information on exposures and potential confounders	Table 1	
		(b) Indicate number of participants with missing data for each variable of interest	Table 1	
		(c) *Cohort study*—Summarise follow-up time (eg, average and total amount)	11	No patient was lost to follow-up for the primary outcome and 30-day mortality. (…)
Outcome data	15*	*Cohort study*—Report numbers of outcome events or summary measures over time	Figure 2	
		Case-control study—Report numbers in each exposure category, or summary measures of exposure	NA	

		Cross-sectional study—Report numbers of outcome events or summary measures	NA	
Main results	16	(*a*) Give unadjusted estimates and, if applicable, confounder-adjusted estimates and their precision (eg, 95% confidence interval). Make clear which confounders were adjusted for and why they were included	9-11, Table 2, Figure 2, Figure 4	
		(*b*) Report category boundaries when continuous variables were categorized	Table 1	
		(*c*) If relevant, consider translating estimates of relative risk into absolute risk for a meaningful time period	NA	
Other analyses	17	Report other analyses done—eg analyses of subgroups and interactions, and sensitivity analyses	10-12, Table 2	No subgroup, interaction, or sensitivity analyses were performed. Secondary outcomes were evaluated, including ICU length of stay, duration of mechanical ventilation, and duration of vasoactive agent administration. Use of eIg was associated with a significantly longer ICU stay and a prolonged duration of mechanical ventilation. No significant Average Treatment Effect was observed for the duration of vasoactive agent administration.
**Discussion**				
Key results	18	Summarise key results with reference to study objectives	Table 2, 12	Our study suggests that administering IgM-enriched IVIg (eIg) may reduce ICU and hospital mortality in surgical patients with sepsis and septic shock.
Limitations	19	Discuss limitations of the study, taking into account sources of potential bias or imprecision. Discuss both direction and magnitude of any potential bias	14	Although its accuracy, our statistical analysis has some limitations. Although IPW techniques performed can "mimic" randomization, only randomization ensures balance for both measured and unmeasured (or unknown) factors. Propensity score methods can only

				adjust for known variables and cannot account for missing data. Post-estimation analysis shows an incomplete Overlap. This suggests that outcome may be influenced by other confounding variables that were not detected and, therefore, not included in our analysis. (there might be confounding factors influencing patients' outcome that were not detected and not measured in our statistical model). Furthermore, the retrospective nature of this study and the limited number of patients enrolled reduce the power of the statistical analysis.<br>In our study no immunological markers were assessed. Information about the immunological condition of the patients, such as circulating immunoglobulin levels, would have been very useful for patient selection and for evaluating the effectiveness of the treatment in restoring immunoglobulin depletion. Additionally, the correlation between outcomes and the kinetic of plasma immunoglobulin values during treatment might provide valuable insights into their relationship.<br>Several studies have shown that immune imbalance can persist in survivors due to long-lasting immune-suppression, leading to the need for hospital readmission and to late mortality. Our study did not include a follow up after Hospital discharge to detect potential reinfections, hospital readmission, or late deaths.
Interpretation	20	Give a cautious overall interpretation of results considering objectives, limitations, multiplicity of analyses, results from similar studies, and other relevant evidence	13-16	Despite its specified limitations, our study suggests, according to other published research, that administration of IgM-enriched IVIg (eIg) could reduce ICU and Hospital mortality in surgical

				patients with sepsis and sepsis shock. Conversely, in our experience, the duration of mechanical ventilation and IVU length of stay were longer in the eIg group, likely due to a more severe clinical condition. The duration of vasopressors does not appear to be influenced by the treatment. With this study we aim to provide encouraging data supporting the use of IgM enriched IVIg for treatment of surgical septic patients. Further studies are needed to confirm our findings and to better understand the role of the IgM-enriched IVIg in treating sepsis. We acknowledge our small sample size; however, as emphasized by Hernán et al. [2021],"…the goal of observational causal inference is not simply to detect effects but to provide transparent estimates. Even imprecise estimates contribute to the evidence base and may guide future meta-analyses… ".
Generalisability	21	Discuss the generalisability (external validity) of the study results	14-15	The results of our study should be cautiously applied to other medical or non-surgical populations.
**Other information**				
Funding	22	Give the source of funding and the role of the funders for the present study and, if applicable, for the original study on which the present article is based	16	No external funding was received. The authors declare no conflicts of interest. Funders had no role in study design, data collection, analysis, interpretation, or manuscript writing.

Journal of Clinical Medicine

MDPI

Supplemental Figures Legends

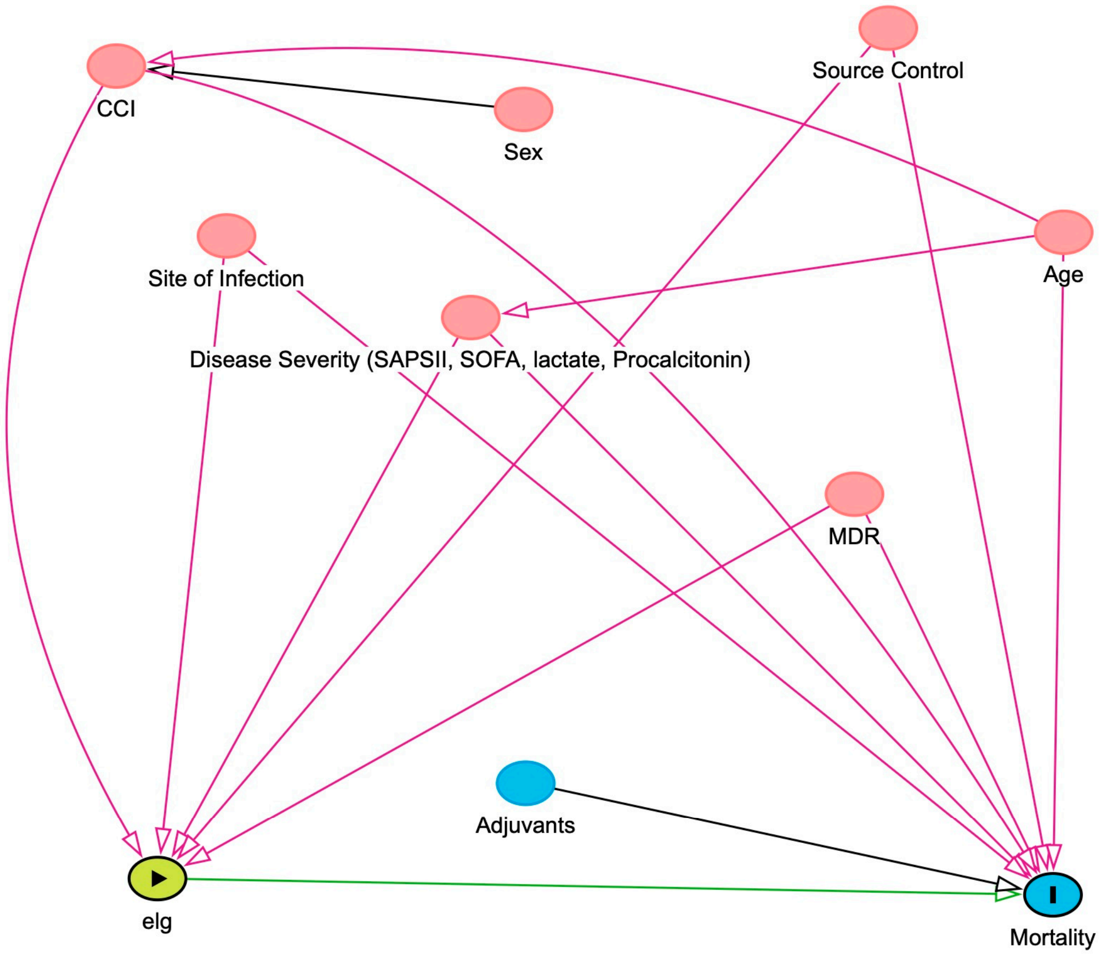

Supplemental Figure S1. Directed acyclic graph (DAG) depicting variables of interest (dots) and causal relantionship between the exposure (treatment with eIg) and the primary outcome (ICU mortality), and the baseline covariates that affects this relationship. SOFA = sequential organ failure assessment score. Dot in blue: primary outcome. Green dot: exposure. The relationship of interest in this study is the one connecting both points. In red: predictor variables that affect both the exposure and the outcome in which direct data was available. In light grey: the presence of complications could influence the outcome but not the treatment received, hence it was not adjusted for.

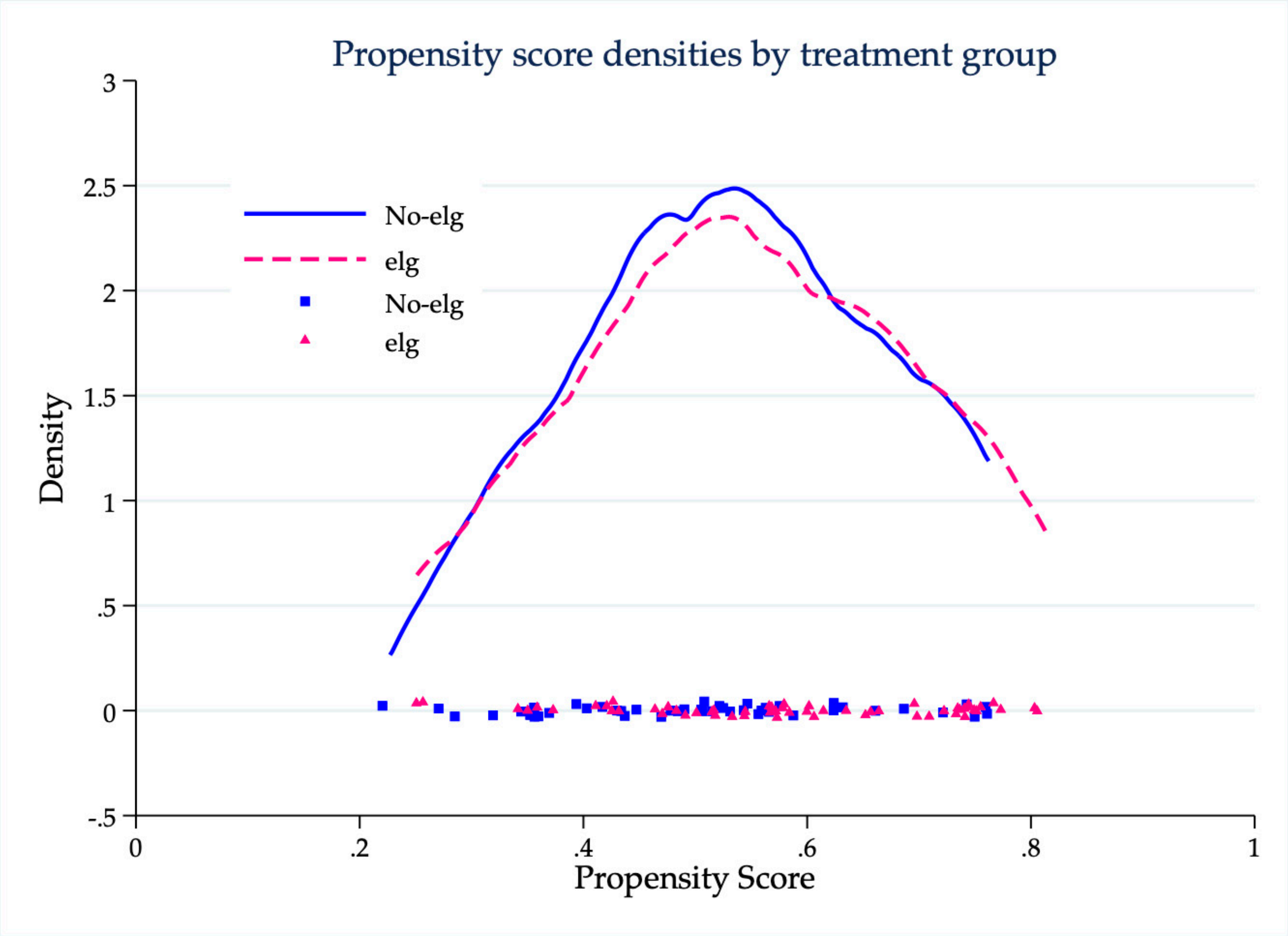

Supplemental Figure S2. The graph displays the estimated density of the predicted probabilities that a patient who re-ceived eIg is classified as a no-eIg patient and the estimated density of the predicted probabilities that a patient who did not receive eIg is classified as an eIg patient, after applying stabilized IPTW. Neither distribution shows excessive probability mass near 0 or 1, and the two estimated densities have most of their respective mass in regions in which they overlap. Thus, there is no evidence that the overlap assumption is violated.
